# Upholding dignity during a pandemic *via* Twitter

**DOI:** 10.12688/f1000research.129829.1

**Published:** 2023-02-16

**Authors:** Michael Mulvey, Tracey O'Sullivan, Sarah Fraser

**Affiliations:** 1 LIFE Research Institute, University of Ottawa, Ottawa, Ontario, K1N 6N5, Canada; 2 Telfer School of Management, University of Ottawa, Ottawa, Ontario, K1N 6N5, Canada; 3 Interdisciplinary School of Health Sciences, University of Ottawa, Ottawa, Ontario, K1N 6N5, Canada

**Keywords:** discourse analysis, social media, text analytics, human rights, vulnerability

## Abstract

**Background:** This article investigates how people invoked the concept of dignity on Twitter during the first year of the COVID-19 pandemic, with a secondary focus on mentions of dignity in the context of older adults and ageing. **Methods:** We report the results of a study that combines text analytic and interpretive methods to analyze word clusters and dignity-based themes in a cross-national sample of 1,946 original messages posted in 2020. **Results:** The study finds that dignity discourse on Twitter advances five major themes: (a) recognize dignity as a fundamental right, (b) uphold the dignity of essential workers, (c) preserve the dignity of at-risk populations, (d) prevent cascading disasters that exacerbate dignity's decline, and (e) attend to death, dignity, and the sanctity of life. **Conclusions:** Moreover, messages focusing on older adults lamented the disproportionate death toll, the terrible circumstances in long-term care homes, the added impact of suspended meal delivery services and the status of older people living below the poverty line.

## Introduction

Dignity is a concept considered by many to be a fundamental human right, reflected in many legal and ethical frameworks around the world, including the Universal Declaration of Human Rights, which states that “all human beings are born free and equal in dignity and rights” ( United Nations, 1948). Those who uphold dignity as a human right often argue that it is an essential component of a just and fair society and should be protected and upheld by governments and other institutions.

This paper investigates how people on social media perceive and express their views on human dignity in the context of the COVID-19 pandemic. To do this, we analyzed a large sample of Twitter posts and comments from the first year of the pandemic to understand how people respond to situations that may involve the violation of dignity. Many studies have shared insight into dignity, drawing perspectives from fields including law ( Cunningham, 2020), public policy and politics ( Nilsson *et al.*, 2022), bioethics ( Faneye, 2014), and healthcare ( Fernández-Sola *et al.*, 2012). However, studies usually focus on expert views, not how the general public expresses thoughts about dignity.

Examining data from social media has many advantages, as it provides unprompted real-time snapshots of how people think and feel about a topic ( Belk, Fisher, and Kozinets, 2013; Berger *et al.*, 2020). It also provides a large and diverse user base from different backgrounds, ages, and locations. Moreover, access to a wide range of archived data (including text, images, and videos) can deliver a large sample of data that is: timely, convenient, cost-effective—and, most importantly—rich and nuanced insights into perceptions. Organizations can then use this information to formulate policy, improve their products and services, and develop more effective marketing and communication strategies.

In the following sections, we describe the theoretical foundations of our approach and report the results of a study of how people talked about dignity on Twitter during the pandemic. Finally, we discuss the value of studying public perceptions of dignity and how crowdsourced views on dignity may inform policies and practices that aim to promote and protect dignity in various settings, such as workplaces, healthcare facilities, and other social settings.

## Theoretical background

### Dignity

While it is clear that dignity and human rights are closely associated, it is important to consider that dignity encompasses: how a person feels (their self-worth) and the context in which they live ( *i.e.*, psychological, social and cultural factors) ( Mégret & Hoffmann, 2009). Furthermore, dignity is socially constructed as it develops and is dependent on social interaction between individuals or groups, and it is also determined by and intersects with other human rights ( Mégret & Hoffmann, 2009). For these reasons, it is likely that individuals or groups that are already marginalized or considered 'vulnerable' ( *i.e.*, older adults, 2SLGBTQI+, Indigenous people, immigrants, *etc.*) are at a greater risk of losing their dignity and to be treated as “not human or less than human–as a thing or instrument or subhuman creature” ( Kateb, 2014). Indeed, in the context of COVID-19, initially, the discourse surrounding older adults living in long-term care homes focused heavily on the loss of dignity in healthcare and in dying ( Carrieri *et al.*, 2020; Colombo, 2021; Kowsalya & Sundara, 2021, Vellani *et al.*, 2021). In general, much of the discourse was ageist, promoting notions that all older adults need protection, are vulnerable and should be socially isolated ( Fraser *et al.*, 2020, Lagacé *et al.*, 2020) –all factors which infringe on human rights and dignity. The impact of COVID-19 on human dignity has been negative, but it has also raised awareness about dignity and the importance of including it as a priority in health system reform ( Galea, 2021).

### Social media and marketplace sentiment

Sociologist Bruno Latour (2007) examines how people and groups within a society make sense of the world and communicate their understanding to others. He studies how language and other forms of representation shape social interactions and relationships, and how they influence the way people construct and communicate their worldviews. Similarly, researchers who examine media discourse and public opinion also focus on the symbols and reasoning devices used to present and discuss issues, such as metaphors, exemplars, catchphrases, depictions, and visual images, as well as the roots, consequences, and appeals to principle that suggest how to think about an issue ( Gamson and Modigliani, 1989).

With increasing convergence of online and offline realms, digital methods of studying communication behaviour have become helpful in gaining insight into people's experiences ( Caliandro, 2018) and sentiments or shared emotional dispositions ( Gopaldas, 2014). As a result, researchers have increasingly turned to social media conversations to understand what people discuss and express. Users post messages on social media that offer a “window into experience” as they share their expertise, exchange questions or concerns, and reinforce each other's positions ( Eriksson and Salzmann-Erikson, 2013). This approach has the advantage of “listening in” on conversations and avoiding certain response biases that may occur when asking questions in interviews or surveys ( Rappaport, 2011).

Applications of social listening in public health and well-being contexts include studies of patients' reactions to knee replacement surgery ( Pitt, Mulvey, and Kietzmann, 2018), gamblers' reflections on problem gambling ( Brown, Caruna, Mulvey, and Pitt, 2021), expressions of stigma against people with dementia ( Bacsu *et al.*, 2022), public attitudes towards vaccines ( Fazel *et al.*, 2021), job satisfaction and turnover ( Lam, Mulvey, and Robson, 2022), and retirement travel planning ( Mulvey, Padgett, and Lever, 2022), using diverse online data sources including discussion forums, review platforms, Facebook, Twitter, and Reddit. In addition, social media methods are well-suited to collecting and analyzing data rapidly in times of crisis or responding to emerging trends, such as the COVID-19 pandemic ( Picone *et al.*, 2020; Reid and Duffy, 2018). Furthermore, advice sharing on social networks is inherently social–people exchange ideas, solicit and deliver advice, and develop relationships with other community members ( Kozinets, 2002; Mulvey, Padgett, and Lever, 2022). Like others, we contend that crowdsourcing holds enormous potential to identify problems and share solutions in times of crisis. For example, self-help groups and advice on social media may improve affected populations' resilience during a disaster, “replacing their helplessness with dignity, control, as well personal and collective responsibility” ( Keim and Noji, 2011).

## Methods

Our study examined original messages from Twitter users, a highly inclusive, one-to-many platform where people share information in a relatively synchronous or condensed time frame ( Lamberton and Humphreys, 2022). Twitter data gives researchers a window into the volume, sentiment, and expressions of public opinion. We conducted a three-step analysis using data from the Sprout Social COVID-19 database, which holds millions of tweets related to the pandemic. First, we examined key performance metrics of the volume and impact of dignity-related tweets to assess the prevalence of dignity-related tweets in 2020. Second, we downloaded a sample of Twitter data *via* Sprout Social and used the Leiden community detection algorithm to detect and study themes in dignity discourse, drawing exemplar tweets to illustrate constituent meanings. Finally, we analyzed themes in the subset of dignity tweets that mention older adults. Ethical approval was waived by the University of Ottawa REB on the basis that data that is publicly available on social media and can be viewed by anyone would not require ethics approval. Our methodology is reproducible and should replicate the reported results, yet a different random sample of tweets may yield slightly different results.

Our cross-national sampling plan aimed to include diverse viewpoints and prevent a single country's tweets from dominating the discourse. Therefore, the sample comprised English-language tweets from countries that posted more than 500 tweets in the 12 months of January to December 2020. Seven countries achieved this level of activity. Also, we decided to prioritize tweets with engagement and followers as opposed to those having little or no impact. So, we sorted the posts in descending order on these metrics before downloading samples proportional to each country's total tweet volume. As a result, the sample includes a total of 1,946 original messages drawn randomly from Twitter, scaled to the United States (1,000 messages), India (300), the United Kingdom (286), Canada (157), South Africa (77), Kenya (65), and Australia (61).

The Leiden community detection algorithm ( Traag, Waltman, & Van Eck, 2019) is a state-of-the-art network-based algorithm that can identify clusters of related nodes in a network. In the context of social media data, we use this algorithm to identify groups of concepts that interact with each other in a meaningful way, such as words co-mentioned in sentences and reflecting latent topics or themes.

We used the Social Astronomy app ( Belanger, 2022) to create a network of words (or n-grams) based on their interactions in social media messages ( *e.g.* words are mentioned together in a tweet). Next, Social Astronomy analyzed the network (or matrix) using the Leiden community detection algorithm to identify clusters of words that interact consistently and cohesively. These clusters may correspond to groups of words that refer to topics or themes. Cluster scores assigned by the algorithm helped retrieve original messages for review by research team members.

The Leiden community detection algorithm results depend on the researcher's selection of resolution parameter setting, which controls the detected communities' granularity and the number of words included in the analysis. A higher resolution parameter will generally detect smaller, more fine-grained communities, while a lower resolution parameter will result in larger, coarser communities. Similarly, including many words will produce a more complex solution than using fewer words. In general, it is helpful to experiment with different values of the resolution parameter to see which one produces the best results given the dataset and analysis objectives.

To improve the accuracy of group membership detection, we ignored stop words (such as “the”, “a”, “an”, and “in”) and ubiquitous words, including the search keywords (dignity, #dignity), a quotation acronym (qt), and Sprout Social's COVID-19 database inclusion keywords (covid, covid19, #covid19, #coronavirus, corona, virus, coronavirus, covid-19, pandemic, #covid, #covid_19, #pandemic, #covid19uk). Mindful of our objective to report results with high fidelity to the original data, we iteratively generated solutions using different settings before selecting a comparatively detailed and stable 13-cluster solution that used the top 200 words and a 1.5 resolution parameter setting.

## Results

Next, we present the listening insights derived from an aggregate analysis of dignity tweets followed by an analysis of themes and topics that emerge from the messages. Social media studies often include quotes to give participants a voice, illustrate ideas to build trust, foster replicability, or lend evidence for claims. However, evolving ethical standards attend to the unintended consequences of reporting individuals’ tweets and express concern about the discoverability of messages posted voluntarily in public forums ( Mason and Singh, 2022). Accordingly, we honour individuals’ privacy and do not reproduce user IDs or tweets. In contrast, we refer to companies, organizations, and institutions that use Twitter for public relations to share views with stakeholders.

### Top-level indicators and metrics

**The prevalence and engagement with dignity-based tweets**

The Sprout Social key performance metrics affirm the popularity of dignity in the reporting period of January to December 2020 and establish that the topic was well-established in COVID-era discourse. The keywords (dignity OR #dignity) appeared in 46,420 original messages (not including shared or mentioned), contributed by 41,060 unique authors, generating 2.89 billion impressions and 5.12 million engagements (total likes, dislikes, comments and shares). These values translate to daily averages of 126.8 messages, 112 new authors, 7.9 million impressions, and 13,991 engagements.

Figure A illustrates the volume and engagement levels of tweets from our sample. There are two panels: the top includes all dignity tweets, excluding dignity tweets focused on older adults, depicted in the bottom panel. Comparatively, the volume of dignity tweets focused on older adults is only 4.4% of the total sample. Each circle denotes a tweet, and its size represents the level of message engagement (the total likes, comments, and shares). Most tweets earned low levels of engagement (small dots), whereas some striking exceptions generated exceptionally high engagement (the circles). For reference, the post with the highest level of engagement (=88,390) generated 56,771 likes, 20,174 comments, and 11,445 shares. Notably, Twitter algorithms favour popular posts, amplifying posts with engagement, feeding them into users' streams and generating more impressions.

**Figure A.**
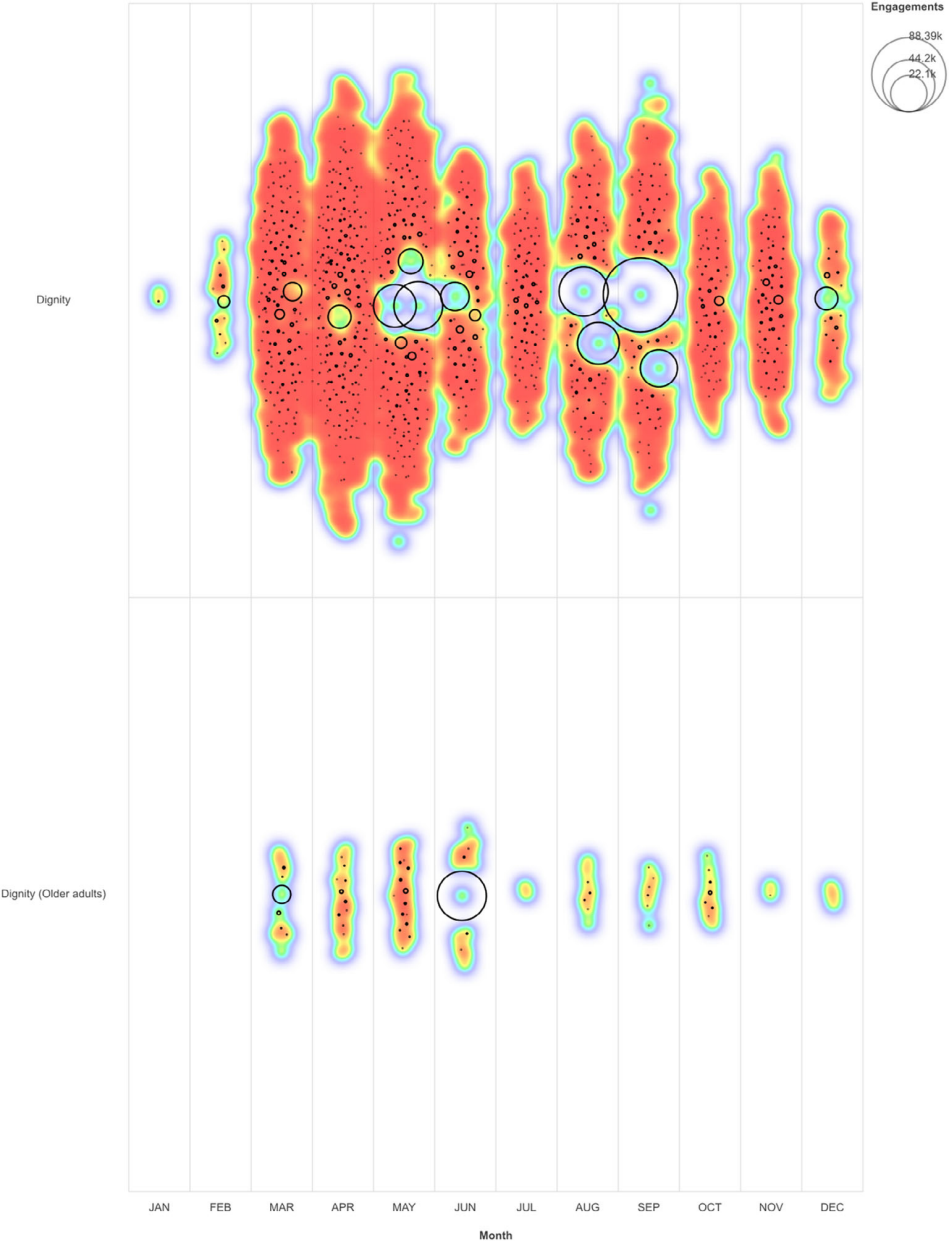
Sample of dignity tweets by month with engagements.

The heat map shading indicates the relative volume of posts by month (red shading represents the highest density, yellow the least). Some observations: dignity discourse on Twitter was sparse in January and February and rose exponentially in March, April, and May following events including the WHO's pandemic declaration, rising hospitalization and death rates, imposition of lockdown measures, and the Black Lives Matter protests. The volume of tweets decreased and maintained steady levels through the summer and fall months and faded into December.

**Tweets with the most engagements**

Next, we focus on the posts that earned top-10 engagement scores. Foremost, politicians championed the cause of dignity, led by a handful of well-known members of the Democratic Party, including Hillary Clinton (#1), Bernie Sanders (#4), Chelsea Clinton (#8, #10), and Nancy Pelosi (#9). In addition, his Holiness Pope Francis (#2, #3, also known as Pontifex), medical doctor Craig Spencer (#5), journalist Miles Howard (#6), and author/retired registered nurse Juanita Broaddrick shared views. These posts demonstrated diverse construals of dignity, invoking tensions between democracy and authoritarianism and respect for persons, including older adults, people who are unemployed, patients and healthcare professionals, small business owners, and leaders (not) doing their duty.

**Core and peripheral words in tweets**

Still, simple word frequency counts and exemplars do not tell the whole story of dignity discourse. Figures B, C, and D include word clouds created using Scimago Graphica that illustrate the relative frequencies of terms, hashtags, and emojis in messages that include the keyword dignity. For example, Figure B shows the prevalence of terms like pandemic and people and negatively charged words such as corruption, authoritarianism, and lawlessness. Figure C indicates how users attach hashtags to messages, linking posts to related conversations, for instance, #blacklivesmatter, #mentalhealth, and #protectreprodignity. Finally, Figure D displays emojis in messages, using visual symbols to convey abstract emotions and feelings, including anger , disgust , and sadness , as well as calls to action to wear a mask , attend to disabled persons ♿, and pray for better outcomes . We also examined mentions of prominent people and brands, signaled by the @ sign, which calls out usernames on Twitter. The list of mentions included the names of many prominent politicians, public health agencies, and news outlets. Increasingly, researchers are attending to hashtags, and emojis as these paratexts that accompany the words may play a prominent role in the reception or interpretation by the public ( Bakker, 2022; Luangrath *et al.*, 2022; Völcker, 2020). By extension, these terms, hashtags, emojis, and mentions suggest a bigger picture, like puzzle pieces, yet they do not form a coherent image.

**Figure B.**
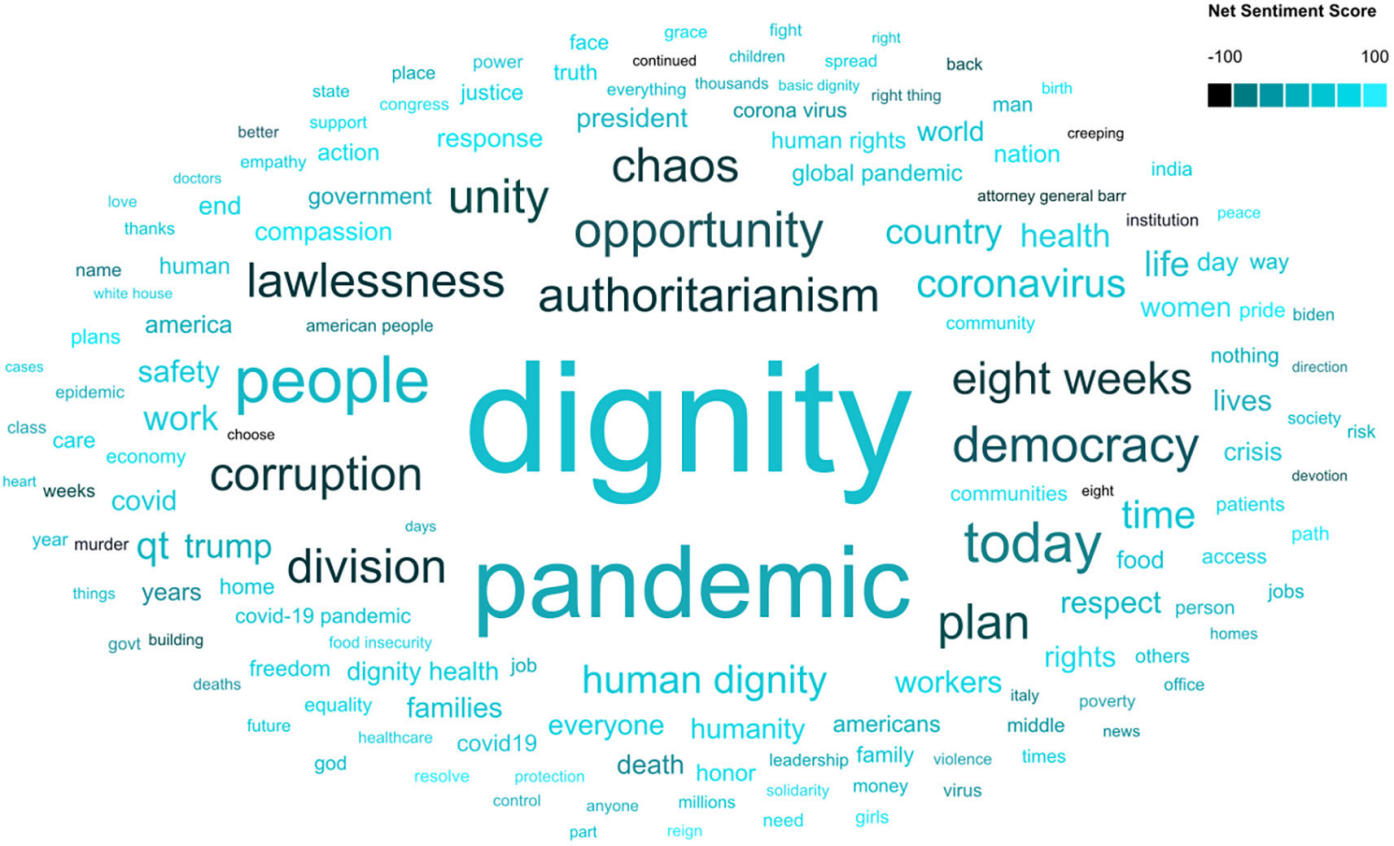
Word cloud of terms in dignity tweets.

**Figure C.**
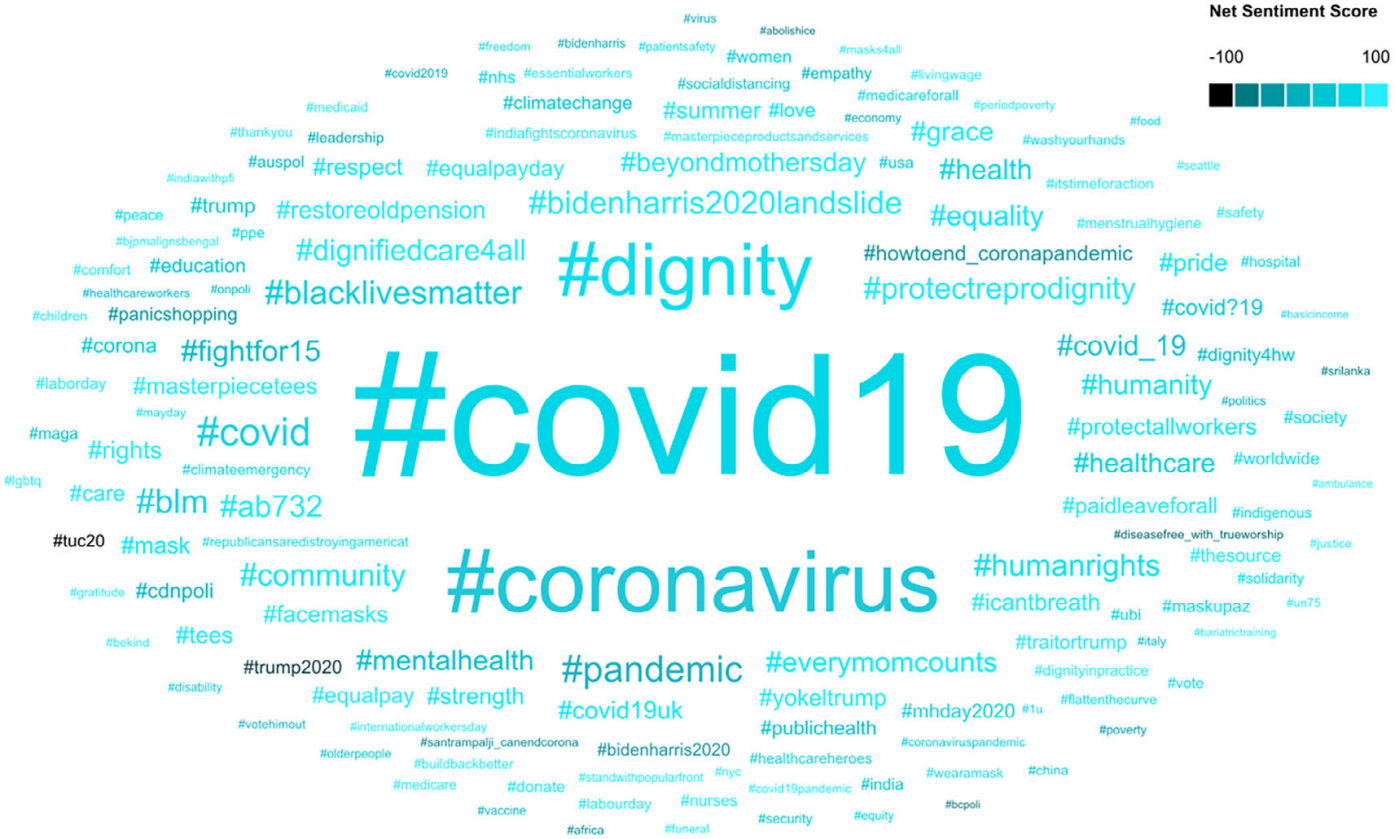
Word cloud of hashtags in dignity tweets.

**Figure D.**
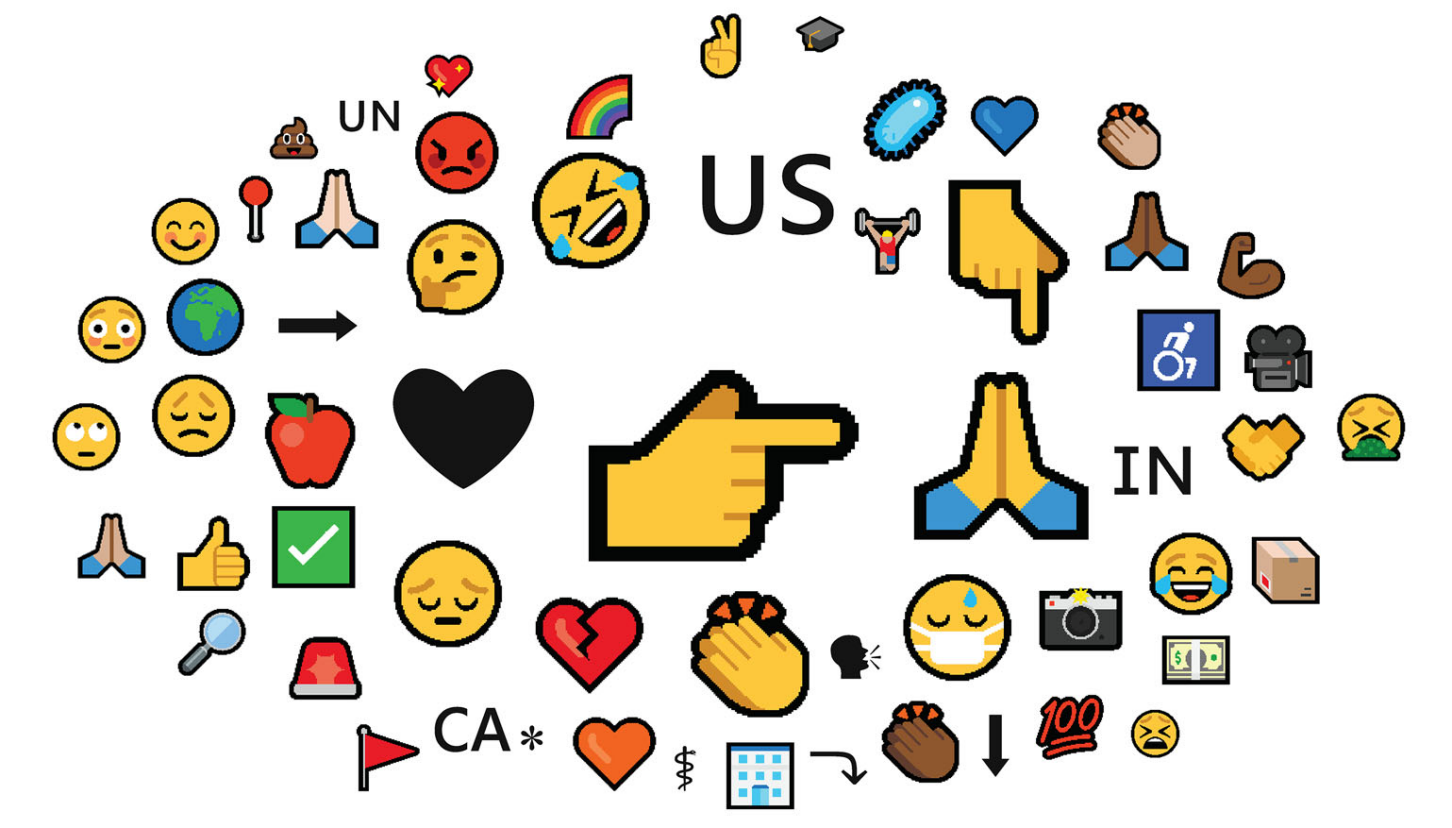
Word cloud of emojis in dignity tweets.

### Thematic analysis and word clusters

Next, our attention turned to analyzing clusters of words used together, focusing on the 200 most frequently used words derived using the Leiden community detection algorithm. Terms of 13 groups form clusters or communities of dignity-based topics in our dataset. Our analysis of these results, combined with iterative readings of the verbatim tweets, helped derive the framework illustrated in Figure E, organizing the thirteen clusters into five broad themes. These words and ideas are typical of the cluster, yet variation exists within clusters, and messages can blend ideas from one or more clusters.

**Figure E.**
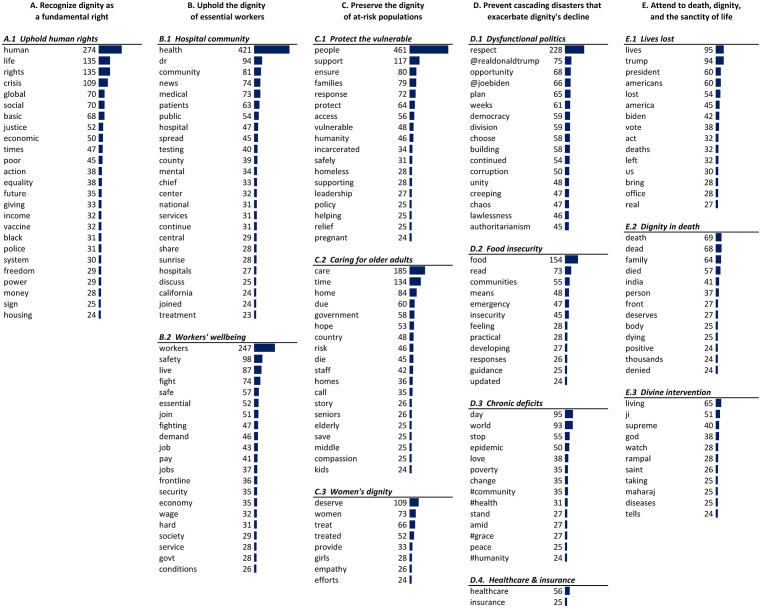
Dignity themes and word clusters.

**Recognize dignity as a fundamental right**

The first theme affirms dignity as a fundamental human right (see Figure E for keywords). Users consider dignity a necessary condition for the enjoyment of human rights, as, without dignity, individuals cannot fully exercise or realize their rights. The global pandemic created crisis conditions that eroded a basic sense of humanity and justice as inequalities in wealth, power, race, and housing conditions threatened individuals' inherent worth and dignity. These inequalities came to the fore with discussions of unequal access to vaccines. For example, one tweet affirmed universal access to vaccines among citizens of South Africa, including migrants.

**Uphold the dignity of essential workers**

The second theme highlights the importance of upholding the dignity of frontline and healthcare workers. The COVID-19 pandemic threatened the dignity of these essential workers in several ways. One such threat is the inadequate provision of personal protective equipment, which put their health and safety at risk. To address this issue, employers must provide workers with the necessary resources and support to carry out their work safely and effectively, including proper equipment, suitable testing measures, adequate training, and sufficient staffing ( The Lancet, 2020; WHO, 2020).

Another way to uphold the dignity of frontline and healthcare workers is by recognizing and valuing their work and contributions. For example, a tweet shared by the Teamsters labour union thanked a member of Local 150 for performing essential work for the Sierra Nevada Memorial Hospital during the pandemic. Employers —and society as a whole—can express gratitude and appreciation for the efforts of these workers by providing them with fair compensation and benefits and offering support and assistance for their physical and mental well-being.

The high stress and isolation many essential workers experienced during the pandemic can lead to burnout, so it is crucial to provide them with the support they need. For example, in a Tweet, the UK-based Living Wage Foundation thanked workers, including school caterers, care workers, cleaners, and delivery drivers, for keeping society going during the pandemic, asserting that they merited a living wage and sufficient work hours to maintain health and live with dignity.

Finally, other messages championed the dignity of frontline and healthcare workers by calling for respect for their rights and dignity as individuals—and avoiding discrimination or harassment. By taking these steps, the public can support and empower essential workers and help them continue providing vital services to society.

**Preserve the dignity of at-risk populations**

The third theme focuses on the close connection between dignity and populations considered vulnerable. For example, India-based WHO South-East Asia issued a tweet that called attention to the need to protect diverse groups in response to the pandemic, including older people, persons with disabilities, people who are homeless, refugees and migrants, people without access to sanitation, and people living in crowded places. Such individuals are more likely to experience disadvantage, discrimination, or exclusion. In addition, due to their limited access to resources, they may struggle with inadequate access to healthcare, education, or social support, which can negatively impact physical and mental health—and undermine dignity and self-worth ( Armitage and Nellums, 2020; Croft and Fraser, 2022). Therefore, it is essential to provide at-risk populations with the necessary support to live decent and fulfilling lives.

One factor that can threaten the dignity of seniors, older adults, and elders is ageism. This discrimination and prejudice can manifest as negative stereotypes, lack of recognition and respect, or unequal access to opportunities and services. As a result, older adults may feel marginalized and devalued, undermining their dignity and self-worth. Another factor that can threaten the dignity of older adults is the inadequate provision of healthcare and social support services–especially during the pandemic. For instance, an individual's sarcastic tweet called out the Liberal–National Coalition (LNP) in Australia for failing to protect the elderly supporters who voted them into power. Other messages submitted that when such services are lacking, older adults may face isolation, loneliness, and loss of independence, all of which can harm their dignity. Additionally, lack of access to adequate housing and financial security can threaten older adults' dignity by limiting their ability to meet their basic needs and participate in society.

Similar to older adults, women may also face unique challenges that can threaten their dignity. These challenges may include pregnancy, parenting responsibilities, domestic abuse, or homelessness, and addressing these issues may require special attention and support. For example, the United Nations Population Fund (UNFPA) Kenya announced a collaboration to provide “dignity kits” to girls living in informal settlements.

**Prevent cascading disasters that exacerbate dignity's decline**

The fourth theme specifies factors that pose a particular challenge to upholding dignity during the pandemic. Critics describe how well-intentioned COVID mitigation strategies suffer from blindspots of missing information about unresolved social problems, neglecting risks, compounding the harm, and leaving dignity in peril.

First, dysfunctional politics can threaten dignity by promoting discriminatory policies and using propaganda and misinformation. Twitter banter between American political parties epitomizes this theme, as a barbed attack between political rivals generated a blunt counter-response with huge engagement. Dignity was a prominent theme in political discourse in the months leading up to the November presidential election. Political strife can lead to political instability and violence (as demonstrated by the Capitol attack on January 6, 2021), so it is crucial to promote functional and inclusive politics that respect the rights and dignity of all individuals.

Second, food insecurity can undermine individuals' dignity by forcing them to make difficult choices and limiting their access to nutritious and healthy food. The COVID-19 pandemic worsened this problem, leading Nourish Scotland to campaign for communities to respond to food insecurity by providing people with access to healthy food options and ensuring social connections.

Third, poverty can undermine individuals' dignity by limiting their access to basic needs and opportunities. Conversely, addressing poverty can help to restore and uphold individuals' dignity by providing them with the means to meet their basic needs and participate in society. Finally, chronic deficits in many domains require a coordinated solution, as the New York-based advocacy group Women Deliver emphasized, calling for world leaders to embrace international collaboration in recovery plans.

Fourth, healthcare and insurance can support and protect an individual's dignity by providing access to necessary medical care and services and protecting them from financial hardship. For example, an individual from Texas tweeted a reminder that coronavirus does not discriminate and that every single person in the country should be able to see a doctor, irrespective of employment or insurance status.

**Attend to death, dignity, and the sanctity of life**

The fifth theme considers the consequences of failing to uphold dignity in the face of death and illness. As the COVID-19 pandemic swept across the world, nations faced a tragedy of unprecedented proportions. Each day, the death toll rose as more people fell victim to the deadly virus. Unfortunately, political tensions—particularly in the United States—plus a lack of a cohesive and effective national response exacerbated the situation. As the tragedy unfolded, it became clear that the country failed to adequately protect its people's dignity. An English political pundit posted an updated death toll, ascribed blame, and urged people to vote.

Respecting an individual's dignity during their end-of-life can be crucial to providing decent end-of-life care. Dignity is a quality or state of being worthy of respect and honour, and as individuals approach the end of life, they often wish for a “good death” that is peaceful, pain-free, and by the individual's wishes and values. Additionally, a Kenyan argued that providing protective suits might allow people to give their final respects to their loved ones with dignity. Finally, some followers of an Indian spiritual leader tweeted claims that they had received a divine cure for the deadly virus, telling of healing without medicine.

### Tweets focused on older adults

Given our interest in examining older adults' challenges in the pandemic, we next examined the subset of tweets that mention older adults. We flagged messages in the sample that included at least one of the following keyword variants: elder* ( *n*=29), senior* ( *n*=28), older ( *n*=24), pension* ( *n*=11), retire* ( *n*=6), or aged ( *n*=1). This procedure netted 86 unique tweets, equal to 4.4% of the sample.

Most messages echoed the dignity themes discussed previously. Foremost, dignity is a human right protected by the United Nations and World Health Organization, a message affirmed by the UN Secretary-General, who reminded world leaders to respect the rights and dignity of older people. Older persons were disproportionately harmed by COVID-19, as reflected in death tolls and social isolation ( Fraser *et al.*, 2020; Sharma, 2021; Wu, 2020). Furthermore, older age intersects with other risk factors, including poverty, disabilities, race, homelessness, immigrants, non-native speakers, and incarcerated persons.

Problems with long-term care homes drew considerable attention as facilities struggled to protect residents from the virus, maintain adequate staffing and care levels, balance social distancing measures, and reduce isolation from family members. The tragic loss of lives sparked calls to hold government officials accountable and to reform the system.

Finally, some seniors' meal delivery services were suspended during the pandemic, placing recipients at risk of not having enough groceries, risking hunger, malnutrition, or even starvation. Poverty increases these risks, as some posts drew attention to low incomes and inadequate pensions or social security benefits.

## Discussion

This study examined social media discourse on dignity posted during the first year of the global COVID-19 pandemic. A secondary goal was to explore the use of dignity in conjunction with ageing. Our cross-national sample, Leiden community detection algorithm and thematic analysis of identified clusters revealed five major themes: (a) recognize dignity as a fundamental right, (b) uphold the dignity of essential workers, (c) preserve the dignity of at-risk populations, (d) prevent cascading disasters that exacerbate dignity's decline, and (e) attend to death, dignity, and the sanctity of life. While each of these themes has distinct contributions, the data reveals that essential workers and at-risk populations were identified as groups whose dignity was heavily impacted during the first year of the pandemic. The implications of the five themes are discussed below.

Tweets in the first three themes reinforced the Universal Declaration of Human Rights to promote dignity as a fundamental human right. In line with Mégret and Hoffman's (2009) seminal work, findings demonstrate the importance of contextual factors and intersection with other human rights. For example, women are more likely than men to be frontline workers in the context of the COVID-19 pandemic ( Utzet et al., 2022) and, as such, face a greater risk of loss of dignity in the context of healthcare provision during a pandemic. The impact on the dignity and health of essential workers has been raised as a target for policy action ( Lancet, 2021). Similarly, the findings of this study align with calls to prioritize the older population, their needs and dignity in times of crisis. Minimizing ageist discourse and maximizing access to healthcare and other services to maintain the health and dignity of this population ( Fraser et al., 2020; Lagacé *et al.*, 2020).

Formal and informal communication—via news outlets and social media—have been essential throughout the pandemic for exchanging information and enhancing awareness of risk and public health recommendations ( Généreux *et al.*, 2020). However, while modern technologies have immeasurable benefits for providing accessible communication, it is also prudent to consider how media discourse can frame issues and shape beliefs during disasters ( Choudhury & Haque, 2018; Wang *et al.*, 2019), including ageist and ableist attitudes ( Barth *et al.*, 2021; O'Sullivan and Phillips 2019). Indeed, Fraser *et al.* (2020) warned of the potential negative impacts of ageist hashtags circulating on social media during the early weeks of the COVID-19 pandemic. Lagacé *et al.* (2021) report similar concerns about the COVID-19 media discourse labelling all older adults as vulnerable people whom we must “fight for” and not “fight along with.” Similarly, Stollznow (2020) describes how prejudice in ageist language perpetuates conflict between boomer and millennial generations, and in the end, impacts everyone.

Our research findings illustrate how social media discourse can be valuable for identifying key issues requiring policy reform. First, the method can amplify the voices of marginalized or underrepresented groups, bringing attention to issues not widely recognized or addressed before. Second, though our analysis was retrospective, the data lends itself to real-time monitoring and rapid response to urgent or pressing issues on social media. Third, it is possible to identify trends and patterns in discussions and debates on social media platforms. Finally, by analyzing the sentiment and engagement with different topics, policymakers can understand what issues are most important to the general public and which issues may create conflict or reduce trust in government or decision-making authorities.

The rise of social media has opened new avenues to explore public opinion, yet it is not without limitations. First, social media users do not represent the general population's views due to self-selection and non-random participation. Therefore, combining this method with other research methods may be necessary to understand public views on human dignity fully. Second, while individuals and organizations have control over the messages they create, the algorithms used by platforms significantly influence the extent to which those messages are seen and received by their intended audience ( Kozinets and Gretzel, 2021). Third, information shared on social media can be contaminated with misinformation and tainted by stereotypes, so it is essential to be vigilant in promoting accurate and credible information and be aware of potential biases.

This study is part of a larger research program focused on how pandemic experiences and exposure to COVID-19 media discourse influenced older adults' perceptions of resilience and vulnerability. It is the first step in a series of research activities where we are exploring how older age and ability are framed in pandemic media discourse. Given the emphasis on dignity in social media, this study provides a glimpse into how the term is generally used in the public sphere and, more specifically, in the context of aging.

As a broader impact, the findings have the potential to inform decision-makers about how the public views dignity and aging. Listening provides feedback on public trust, confidence and priorities and can assist decision-makers by understanding the discourse circulating in social media. Given the conceptualization of dignity as a human right and its relevance for combatting ageism, these findings can help promote healthy ageing throughout pandemic recovery and adaptation to a 'new normal'.

### Data and software availability

The underlying data to this research cannot be shared due to the ethical and copyright restrictions surrounding social media data. The Methods section contains detailed information to allow replication of the study which used the Type, Date, Location, Engagement, and Message fields of archived Twitter data records. Any queries about the methodology should be directed to the corresponding author.

The Social Astronomy app used to analyze clusters is proprietary; contact Belanger Analytics Inc. The software's underlying functions can be executed using open-sourced APIs, such as Scimago Graphica.

## References

[ref1] Armitage R Nellums LB : The COVID-19 response must be disability inclusive. *Lancet Public Health.* 2020;5(5):e257. 10.1016/S2468-2667(20)30076-1 32224295 PMC7270835

[ref2] Belanger C : *Social Astronomy.* Belanger Analytics Inc.;2022.

[ref3] Bacsu J Fraser S Chasteen A : Using Twitter to examine stigma against people with dementia during COVID-19: Infodemiology Study. *JMIR Aging.* 2022;5(1):1–10. 10.2196/35677 35290197 PMC9015751

[ref4] Bakker D : *Tracing the symbolic contours of a lifestyle product category using# hashtags (Masters thesis, Université d'Ottawa/University of Ottawa).* 2022. 10.20381/ruor-28363

[ref5] Barth N Guyot J Fraser SA : COVID-19 and quarantine, a catalyst for ageism. *Front. Public Health.* 2021;9:589244. 10.3389/fpubh.2021.589244 33912526 PMC8072107

[ref6] Belk R Fischer E Kozinets RV : *Qualitative consumer & marketing research.* London, UK: Sage;2013.

[ref7] Berger J Humphreys A Ludwig S : Uniting the tribes: Using text for marketing insight. *J. Mark.* 2020;84(1):1–25. 10.1177/00222429198731

[ref8] Brown T Caruana A Mulvey M : Understanding the emotions of those with a gambling disorder: Insights from automated text analysis. *J. Gambl. Issues.* 2021;47(Spring):121–142. 10.4309/jgi.2021.47.5

[ref9] Caliandro A : Digital methods for ethnography: Analytical concepts for ethnographers exploring social media environments. *J. Contemp. Ethnogr.* 2018;47(5):089124161770296–089124161770578. 10.1177/0891241617702960

[ref10] Carrieri D Peccatori FA Boniolo G : COVID-19: A plea to protect the older population. *Int. J. Equity Health.* 2020;19(1):1–4. 10.1186/s12939-020-01193-5 32430077 PMC7235539

[ref11] Choudhury M-U-I Haque CE : Interpretations of resilience and change and the catalytic roles of media: A case of Canadian daily newspaper discourse on natural disasters. *Environ. Manag.* 2018;61(2):236–248. 10.1007/s00267-017-0980-7 29307042

[ref12] Colombo E : Human rights-inspired governmentality: COVID-19 through a human dignity perspective. *Crit. Sociol.* 2021;47(4–5):571–581. 10.1177/0896920520971846

[ref13] Croft S Fraser S : A scoping review of barriers and facilitators affecting the lives of people with disabilities during COVID-19. *Front. Rehabil. Sci.* 2022;2:119. 10.3389/fresc.2021.784450 36188856 PMC9397712

[ref14] Cunningham S : *Sex work and human dignity: Law, politics and discourse.* Routledge;2020. 10.4324/9780429355424

[ref15] Eriksson H Salzmann-Erikson M : Cyber nursing—Health' experts' approaches in the post-modern era of virtual performances: A nethnography study. *Int. J. Nurs. Stud.* 2013;50(3):335–344. 10.1016/j.ijnurstu.2012.09.014 23040763

[ref16] Faneye B : Human dignity and human rights: A universal language for bioethics. *Philos. Stud.* 2014;4(1):11–19. 10.17265/2159-5313/2014.01.002

[ref17] Fazel S Zhang L Javid B : Harnessing Twitter data to survey public attention and attitudes towards COVID-19 vaccines in the UK. *Sci. Rep.* 2021;11(1):23402–23405. 10.1038/s41598-021-02710-4 34907201 PMC8671421

[ref18] Fernández-Sola C Granero-Molina J Manrique GA : New regulation of the right to a dignified dying in Spain: Repercussions for nursing. *Nurs. Ethics.* 2012;19(5):619–628. 10.1177/0969733011429016 22323394

[ref19] Fraser S Lagacé M Bongué B : Ageism and COVID-19: What does our society's response say about us? *Age Ageing.* 2020;49(5):692–695. 10.1093/ageing/afaa097 32377666 PMC7239227

[ref20] Galea S : Elevating dignity as a goal for health system achievement in the COVID-19 era and in the future. *JAMA Health Forum.* American Medical Association;2021, August; (Vol.2(8): pp.e212803–e212803). 10.1001/jamahealthforum.2021.2803 36218710

[ref21] Gamson WA Modigliani A : Media discourse and public opinion on nuclear power: A constructionist approach. *Am. J. Sociol.* 1989;95(1):1–37. 10.1086/229213 Reference Source

[ref22] Généreux M David M O’Sullivan T : Communication strategies and media discourses in the age of COVID-19: An urgent need for action. *Health Promot. Int.* 2020. 10.1093/heapro/daaa136/6028470 PMC7799077 33294917

[ref23] Gopaldas A : Marketplace sentiments. *J. Consum. Res.* 2014;41(4):995–1014. 10.1086/678034

[ref24] Keim ME Noji E : Emergent use of social media: a new age of opportunity for disaster resilience. *Am. J. Disaster Med.* 2011;6(1):47–54. 10.5055/ajdm.2010.0000 21466029

[ref25] Kozinets RV : The field behind the screen: Using netnography for marketing research in online communities. *J. Mark. Res.* 2002;39(1):61–72. 10.1509/jmkr.39.1.61.18935

[ref26] Kozinets RV Gretzel U : Commentary: Artificial Intelligence: The Marketer's Dilemma. *J. Mark.* 2021;85(1):156–159. 10.1177/0022242920972933

[ref30] Kateb G : *Human dignity.* Harvard University Press;2014.

[ref31] Kowsalya B Sundara Raj T : Elders' dignity and challenges during Covid-19 pandemic. *Indian Journal of Gerontology.* 2021;35(2):314–326.

[ref27] Lagacé M Doucet A Dangoisse P : The “vulnerability” discourse in times of Covid-19: Between abandonment and protection of Canadian Francophone older adults. *Front. Public Health.* 2021;9:1–9. 10.3389/fpubh.2021.662231 34540778 PMC8446363

[ref54] Lagacé M Mérette M Navaux J : An Examination of the Social and Economic Impacts of Ageism. *Employment and Social Development Canada.* 2020. Reference Source

[ref28] Lam J Mulvey M Robson K : Looking through the Glassdoor: The stories that B2B salespeople tell. *Ind. Mark. Manag.* 2022;105(August):478–488. 10.1016/j.indmarman.2022.07.004

[ref29] Latour B : *Reassembling the social: An introduction to actor-network-theory.* Oxford University Press;2007.

[ref32] Lamberton C Humphreys A : Social media: From classic psychological theories to new opportunities. *APA Handbook of Consumer Psychology.* American Psychological Association;2022; (pp.489–511). 10.1037/0000262-021

[ref33] Lancet T : Health and care workers are owed a better future. *Lancet (London, England).* 2021;397(10272):347. 10.1016/S0140-6736(21)00179-3 33516320

[ref34] Luangrath AW Xu Y Wang T : Paralanguage classifier (PARA): An algorithm for automatic coding of paralinguistic nonverbal parts of speech in text. *J. Mark. Res.* 2022;002224372211160. 10.1177/00222437221116058

[ref35] Mason S Singh L : Reporting and discoverability of “Tweets” quoted in published scholarship: current practice and ethical implications. *Research Ethics.* 2022;18(2):93–113. 10.1177/17470161221076948

[ref36] Mégret F Hoffmann F : Dignity: A special focus on vulnerable groups. *Swiss Initiative to Commemorate the 60th Anniversary of the UDHR Protecting Dignity: An Agenda for Human Rights.* 2009.

[ref37] Mulvey MS Padgett DT Lever MW : Sustaining travel dreams in retirement: Guidance at the crossroads. *Well-being In Later Life.* Routledge;2022; (pp.65–81). 10.4324/9781003242468

[ref38] Nilsson M Andersson S Magnusson L : Keeping the older population and their informal carers healthy and independent using digital technology: A discourse analysis of local policy. *Ageing Soc.* 2022;1–31. 10.1017/S0144686X22000514

[ref39] O'Sullivan T Phillips K : From SARS to Pandemic Influenza - The Framing of High-Risk Populations. *Nat. Hazards.* 2019;98:103–117. 10.1007/s11069-019-03584-6 32214659 PMC7088565

[ref40] Picone M Inoue S DeFelice C : Social listening as a rapid approach to collecting and analyzing COVID-19 symptoms and disease natural histories reported by large numbers of individuals. *Popul. Health Manag.* 2020;23(5):350–360. 10.1089/pop.2020.0189 32897820

[ref41] Pitt C Mulvey M Kietzmann J : Quantitative insights from online qualitative data: An example from the healthcare sector. *Psychol. Mark.* 2018;35(12):1010–1017. 10.1002/mar.21152

[ref42] Rappaport SD : *Listen first!: Turning social media conversations into business advantage.* John Wiley & Sons;2011.

[ref43] Reid E Duffy K : A netnographic sensibility: Developing the netnographic/social listening boundaries. *J. Mark. Manag.* 2018;34(3-4):263–286. 10.1080/0267257X.2018.1450282

[ref55] Sharma A : Estimating older adult mortality from COVID-19. *The Journals of Gerontology: Series B.* 2021;76(3):e68–e74. 10.1093/geronb/gbaa161 PMC7543625 32931554

[ref44] Stollznow K : God's Waiting Room. *On the Offensive: Prejudice in Language Past and Present.* Cambridge: Cambridge University Press;2020; (pp.222–250). 10.1017/9781108866637.008

[ref45] The Lancet: COVID-19: protecting health-care workers. *Lancet (London, England).* 2020;395(10228):922. 10.1016/S0140-6736(20)30644-9 32199474 PMC7138074

[ref46] Traag VA Waltman L Van Eck NJ : From Louvain to Leiden: Guaranteeing well-connected communities. *Sci. Rep.* 2019;9(1):1–12. 10.1038/s41598-019-41695-z 30914743 PMC6435756

[ref47] United Nations: Universal declaration of human rights. 1948. Reference Source

[ref48] Utzet M Bacigalupe A Navarro A : Occupational health, frontline workers and COVID-19 lockdown: New gender-related inequalities? *J. Epidemiol. Community Health.* 2022;76(6):537–543. 10.1136/jech-2021-217692 35228295

[ref49] Vellani S Boscart V Escrig-Pinol A : Complexity of nurse practitioners' role in facilitating a dignified death for long-term care home residents during the COVID-19 pandemic. *J. Pers. Med.* 2021;11(5):433. 1-15. 10.3390/jpm11050433 34069545 PMC8161387

[ref50] Völcker M : Paratexts on a social network site and their relevance in the production of meaning—Results of a qualitative investigation of Twitter-Feeds. *Plos One.* 2020;15(9):e0238765. 10.1371/journal.pone.0238765 32946465 PMC7500677

[ref51] Wang Y McKee M Torbica A : Systematic literature review on the spread of health-related misinformation on social media. *Soc. Sci. Med.* 2019;240:112552. 10.1016/j.socscimed.2019.112552 31561111 PMC7117034

[ref52] WHO: *Keep health workers safe to keep patients safe: WHO [Press release].* 2020. Reference Source

[ref53] Wu B : Social isolation and loneliness among older adults in the context of COVID-19: A global challenge. *Global Health Research and Policy.* 2020;5(1):1–3. 10.1186/s41256-020-00154-3 32514427 PMC7272234

