# Supplementary material for: Upholding dignity during a pandemic via Twitter
Source: F1000Res. 2023 Feb 16;12:183. [Version 1] doi: 10.12688/f1000research.129829.1 (PMC10948971; doi:10.12688/f1000research.129829.1)
Supplement: Peer review history

# Reviewer report: Reviewer response for version 1

**DOI:** 10.5256/f1000research.142542.r187216

**Published:** 2024-03-18

**Authors:** Uğur Gündüz

**Affiliations:** 1 Faculty of Communication, Prof. Dr., Istanbul University, Istanbul, Turkey

The paper entitled "Upholding Dignity During a Pandemic via Twitter" provides valuable insights into how people expressed their views on dignity during the COVID-19 pandemic using social media. By analyzing 1946 original tweets using a combination of text analytics and interpretive methods, the study identifies five major themes related to dignity discourse: recognition of dignity as a fundamental right, upholding the dignity of essential workers, preserving the dignity of at-risk populations, preventing cascading disasters exacerbating dignity's decline, and attending to death dignity and the sanctity of life. This paper contributes to the existing literature by highlighting public sentiment on these themes and suggesting how such insights might inform policies and practices to protect and promote dignity. The paper covers an important research area, and overall, the paper is well articulated and presented. However, I would like to see the following concerns addressed:

 So, I recommend minor revision as indicated below:

 1-The paper would benefit from a more detailed explanation of the selection criteria for the tweets included in the study. Specifically, clarity is needed on how the tweets were deemed relevant to the study's themes of dignity, and any exclusion criteria should be explicitly stated.

 2- A brief discussion on the limitations of the interpretive methods used and how they might impact the study's findings would strengthen the research's credibility.

 3-While the identified themes are compelling, the discussion would benefit from a deeper exploration of how these themes interact with each other. For example, how does the recognition of dignity as a fundamental right influence actions towards at-risk populations? Expanding on these connections could offer more nuanced insights into the public discourse around dignity.

 4-The study mentions a cross-national sample but does not delve into regional variations in dignity discourse. Given the global impact of the COVID-19 pandemic, highlighting any geographic differences in the perception of dignity on Twitter could enrich the analysis. If data allows, a comparison between countries or regions might reveal culturally specific concerns or expressions of dignity.

 5-The conclusion section could be expanded to offer more concrete suggestions for future research directions based on the findings. Furthermore, providing specific policy recommendations or interventions to address the issues raised by the study would make the manuscript more impactful. For instance, suggesting ways social media platforms could promote dignity in public discourse during crises could be a valuable addition.

 6-There are minor typographical and grammatical errors that need correction to improve the manuscript's clarity and professionalism.

 Good luck with your research !..

Is the work clearly and accurately presented and does it cite the current literature?

Yes

If applicable, is the statistical analysis and its interpretation appropriate?

Yes

Are all the source data underlying the results available to ensure full reproducibility?

Partly

Is the study design appropriate and is the work technically sound?

Yes

Are the conclusions drawn adequately supported by the results?

Partly

Are sufficient details of methods and analysis provided to allow replication by others?

Yes

Reviewer Expertise:

New Media, Social Networking, Media and Cultural Studies

I confirm that I have read this submission and believe that I have an appropriate level of expertise to confirm that it is of an acceptable scientific standard, however I have significant reservations, as outlined above.

# Reviewer report: Reviewer response for version 1

**DOI:** 10.5256/f1000research.142542.r187199

**Published:** 2024-03-08

**Authors:** Jan Kietzmann

**Affiliations:** 1 Peter B. Gustavson School of Business, University of Victoria, Victoria, BC, Canada

I really enjoyed reading this piece. As a social media researcher, I was mostly interested in the methodology for using Twitter data, as I have seen many different approaches. Here, I was thoroughly impressed by the rigor demonstrated by the authors. In terms of the topic itself, I have to admit that I am completely new to the focus on dignity. I have researched online exploitation of people, in particular exploitations of a sexual nature related to children. In my work, I had not considered the dignity literature, and can now see how much value this angle would add to my own work. Thank you, to all of the authors, for introducing this important lens. It is without a doubt that other readers will learn lots, too. I understand that their focus was on COVID related comments but can see how their approach can be replicated for many other instances, far beyond exogenous shocks like COVID.

 In terms of readability, I would also like to compliment the authors on the attention they paid to ensure that a newbie could easily consume and understand this deep and important topic. Likewise, the methods section was so clear that others, interested in running a Twitter study themselves, could now replicate the methods employed.

 As far as Twitter data is concerned, the platform comes with the usual pros and cons, and I was happy to see that the authors acknowledged this. As a non-native speaker, I am keenly aware of the dominance of English on Twitter and social media in general. After having lived and/or spent a lot of time in many of the countries that were included, I was curious if one should try to correct for the cultural and linguistic differences between the countries included. I am not convinced that this would strictly speaking be necessary, but the authors could add a short reference to other articles that have used similar geographically diverse settings etc.

 I was looking forward to the discussion, and once again, was impressed with how all of the pieces were connected here. Well, done. The one point I noted was that I was not clear on who the main audience of this piece was. Is it public policy makers? If so, the focus can be sharpened from the beginning to the end. If the audience is broader, maybe a simple table that outlines what different segments can take away from this study would be interesting. Please do not see this as a strong point of criticism, but as food for thought. 

 As you can see, I really, really enjoyed this piece and have nothing that I think I'd change. It's a narrowly focused, rigorously executed and superbly documented research study. 

 As a complete aside, I enjoyed the emoji word cloud - very interesting. I will now have to learn how to interpret emojis in a rigorous way :-).

Is the work clearly and accurately presented and does it cite the current literature?

Yes

If applicable, is the statistical analysis and its interpretation appropriate?

Not applicable

Are all the source data underlying the results available to ensure full reproducibility?

Yes

Is the study design appropriate and is the work technically sound?

Yes

Are the conclusions drawn adequately supported by the results?

Yes

Are sufficient details of methods and analysis provided to allow replication by others?

Yes

Reviewer Expertise:

Social media research

I confirm that I have read this submission and believe that I have an appropriate level of expertise to confirm that it is of an acceptable scientific standard.

# Reviewer report: Reviewer response for version 1

**DOI:** 10.5256/f1000research.142542.r208735

**Published:** 2023-10-13

**Authors:** Meiko Makita, Hannah Loret

**Affiliations:** 1 School of Health Sciences, University of Dundee, Dundee, Scotland, UK; 2 School of Health Scieces, University of Dundee, Dundee, Scotland, UK

**Comments to the authors by reviewer 1 (Meiko Makita)**

 The paper reads fairly well and offers insights into how dignity was perceived and discussed during the early days of the pandemic, particularly in relation to older adults and various societal concerns. I believe the manuscript can be further improved. Here are some revisions I suggest for strengthening the overall quality of the manuscript.

 1. Form and style.

- Please be consistent with either British or American language. Throughout the manuscript you use both (e.g., ageing, aging).
- In-text citations are presented in at least two different formats.
- Please review the references list.
- On page 3, par 5: (i.e., older adults, 2SLGBTQI+, Indigenous people, immigrants, etc.) it should be ‘e.g.’ without ‘etc.’. t
- The acronym LGBTQI+ is well-known, however I do not know what 2S stand for, perhaps it would be better to spelled it out.

2. Section Theoretical Background/Social Media and Marketplace sentiment

 I was surprised the authors only covered very broad social media studies and did not include any study on older adults/ageism and social media, such as the two I shared below,

 Reuben Ng, Nicole Indran, Hostility Toward Baby Boomers on TikTok, *The Gerontologist*, Volume 62, Issue 8, October 2022, Pages 1196–1206, https://doi.org/10.1093/geront/gnac020

 Elliott, R. (2022). The ‘Boomer remover’: Intergenerational discounting, the coronavirus and climate change. The Sociological Review, 70(1), 74-91. https://doi.org/10.1177/00380261211049023

 3. Methodology

 The method appears to have followed a systematic approach and the authors state that their study is reproducible, which is a positive aspect in terms of rigour. In the Discussion section the authors mention:  “Finally, by analyzing the sentiment and engagement with different topics, policymakers can understand what issues are most important to the general public and which issues may create conflict or reduce trust in government or decision-making authorities.”

 However, no sentiment analysis of the tweets data set was conducted. Could you please either conduct a proper sentiment analysis or explain what is that you mean by ‘analysing the sentiment’?

 I suggest moving the text that is currently in the first paragraph of Results (below) to the Methods section, when ethics is introduced:

*Social media studies often include quotes to give participants a voice, illustrate ideas to build trust, foster replicability, or lend evidence for claims. However, evolving ethical standards attend to the unintended consequences of reporting individuals’ tweets and express concern about the discoverability of messages posted voluntarily in public forums (Mason and Singh, 2022). Accordingly, we honour individuals’ privacy and do not reproduce user IDs or tweets. In contrast, we refer to companies, organizations, and institutions that use Twitter for public relations to share views with stakeholders.*

 I’d also further elaborate on the ethical implications of using publicly available online data. There is a lot of debate on this issue and the authors should engage with this literature and further discuss their stance on using publicly available online data.

 Please see for instance: Stommel W, Rijk L de. Ethical approval: none sought. How discourse analysts report ethical issues around publicly available online data. Research Ethics. 2021;17(3):275-297. doi: 10.1177/1747016120988767

 The method employed is not discourse analysis but thematic analysis. This is also supported by the name of the subsection ‘thematic analysis and word clusters’. Perhaps remove from the keywords ‘discourse analysis’ and only retain ‘discourse’.

 4. Results

 Tweets with most engagement – is this about number of likes and retweets? Could we have more data on this for each of the top-10 accounts?

 Also, since the audience would be not only from North America, probably best to specify this is USA’s Democratic Party.

 In comparison, with the other four themes, ‘recognise dignity as a fundamental right’ is not as developed. Or is the first theme the overarching theme and the other four subthemes?

 Consider changing the themes to an ‘ing’ verb form, example: Preserving the dignity of at-risk populations.

 The section ‘Tweets focused on older adults’ seems to be a little underdeveloped, especially since is a focus of the study. In here, the authors could perhaps introduce examples of what was said in tweets, as they did with the other themes.

 5. Discussion

 Once the authors expand on their theoretical background section, they would be able to also strengthen their discussion.

 The concept of ‘healthy ageing’ is only introduced at the end of the manuscript – if this is important try to bring it up much earlier on (intro, background).

 Consider adding a ‘conclusion’ section.

**Comments to the authors by reviewer 2 (Hannah Loret)**

 Title and Abstract:

- The summary of the background and purpose of the research should better articulate the research question and the need for this study to have been undertaken.
- Sample size is clear, but how the data was identified and sourced could be covered here.
- The conclusion is very descriptive. What is the main finding (it is clear however why we should lament the unacceptable situation for older adults)? This could show a stronger critical engagement with the results of the study.

Introduction:

- Referring to structurally underserved ‘vulnerable’ people as ‘creatures’ (p. 3 para 5) could be better expressed here, by choosing a different quotation to make the point. As it stands, the way it is currently expressed risks repeating the terminology used to marginalise the groups mentioned.
- Later in this same paragraph, there is the claim that to consider older people as all vulnerable is ageist, but earlier in the paragraph the authors point to older people as a vulnerable group. Perhaps this could be mitigated by suggesting older people, ‘immigrants etc.’ are *potentially *vulnerable, allowing for slightly more nuanced use of the term?
- P. 3 paragraph 6 I am unsure why Latour is being brought in. Are his methods being used? This reference to his work or approach doesn’t seem to be upheld in this section or signposting to the methodology, beyond saying that research into media discourse and public opinion (which are two different things – perhaps a distinction could be made?) has a similar investment in making meaning.
- (P.4) I would like to see more references and conclusions drawn in this field which justify the need for the study from sources which aren’t written by this article’s authors.
- I understand on a basic level why this approach has been chosen, but I understand less what the current literature in this specific field looks like and what contribution to the field this article makes. It could be much more explicit in the theoretical background exactly why this study was needed from both a theoretical and empirical perspective.
- I’m unclear from the introduction what the exact aims or hypotheses of this study/article are. Can these be stated explicitly?
- Why were older adults chosen over any other demographic group for the focus here? Please expose the gap in the literature and the rationale for this choice.

Methods:

- (Para 2, p. 4) I disagree that Twitter is a highly inclusive platform. What about people who have literacy issues or with a lack of digital skills, internet access, or other unmet access needs which prevent them engaging with it?
- (Para 3, p. 4) why were English-language tweets chosen over another language? The rationale should be clearer for this.
- (P. 4) can you speak a little more about how this data was analysed, to signpost to the results section? The methods used are clear and seem appropriate, but I’m struggling to see how they fit within the overall aims of the project.
- (p. 4) could a rationale be given for why this method was chosen over another, and how any bias may function within it?
- I find the design coherent, but the research questions need to be more clearly stated earlier in the article so that perhaps the methodology section conveys how these will be answered more clearly.
- Some of the information in the ‘results’ section would be better placed in the ‘methodology section’, as it is describing the methodology rather than the results. I’m particularly thinking of the last section of the final paragraph on page 5 where this is a description of methodology rather than results. Methodological explanations at the beginning of the results section could be incorporated into the methodology, allowing more room for the thematic structuring of the results sections.

Results:

- A short summary of the main results which relates to the research questions (as well as methodological focus) would be helpful.
- I particularly like the final paragraph of page 5. The tone is pleasantly authoritative.
- (P.8, final paragraph) I’m unsure why migrants in South Africa have been chosen to demonstrate this point. Could this point be made clearer with reference to the points in the preceding sentence, for example by explicitly explaining how this relates to questions of race/wealth/housing conditions?
- (p.9, para 4) I’m unclear how the study data demonstrates that by ‘taking these steps, the public can support and empower essential workers and help them continue providing vital services to society’. My understanding is that the data demonstrates a public acknowledgement and will to improve the working conditions of essential workers but the steps to this are unclear, and it is unclear how a Twitter discourse actively empowers workers or helps them to deliver services. Perhaps a closer reading of the data gathered could make this clearer, in terms of how the public demonstrated support for the dignity of essential workers through their tweets, then linking this to any related targeted activism which followed for those particular groups of working people?
- (p. 9, para 5) This section could also benefit from some closer reference to the empirical data from the study to strengthen its statements and enrich the points made. I also questioned the use of the term ‘at risk’ when the term vulnerable had been used before. Perhaps it would be good to stick to one of these terms or to give a reason for the change in terminology?
- (P. 9, para 6) Is there a reason that this section chooses to focus more closely on older adults as a structurally underserved population? This needs to be more explicit.
- (P.9, para 7) The first sentence here makes it sound like older adults and women are two completely distinct groups, and that older women are not included in older adults. Please reword to clarify that older adults includes women, though (younger?) women experiencing domestic abuse, precarious housing, pregnancy-related issues etc. may be affected by unique gender-related challenges.
- (P.9, para 8) The paragraphs here are very short and therefore lose a sense of coherence. They could be joined without losing the sense of the argument, and this would also help to strengthen the use of empirical data to evidence the points being made (I would again like to see more information from the empirical data to back up the statements being made here).
- (P.9, para 8) Needs a reference or several references for ‘critics describe…’
- (p. 10, para 1) Readers who are not in the USA may not be familiar with the political parties or political structure there, or the exact details of the Capitol attack in January 2021. If this is to be included, it will need to be qualified by some more information about the USA political system which links to the point being made about propaganda/misinformation and discriminatory policies.
- On this point, I think discriminatory policies and propaganda/misinformation are two separate points. Perhaps these could be teased out briefly with reference to empirical data to justify these statements.
- (P. 10, para 4) It is important to acknowledge that although many healthcare systems operate both publicly and privately, and have social security, healthcare system design varies and the example given from Texas will be unique to the case in the USA which should be clearer – for example in Scotland, ‘insurance status’ would mean something very different in terms of healthcare, and most of the population is able to access primary and secondary care free of charge.  
- (p. 10, para 6) it is unclear why we are suddenly speaking about end-of-life care. This could be better linked to the empirical data, and could be linked to the above paragraph with a signposting sentence to improve the flow of the article.
- (p. 10, para 6) It is unclear why we are looking at Kenya and India for these mentions, the connection could be more explicit and better linked back to the concerns of the overall analysis (if the article wants to concentrate on meaning, there will need to be more critical appraisal or expansion on possible meanings here as elsewhere).
- It is not until p. 10 we are told why older adults are a particular focus. This rationale is strong and needs to figure much earlier in the article.

Figures and Tables:

- I find that figures and tables are clear, easily interpreted, and well-formatted.

Discussion:

- The start to this section is strong, and the tone is appropriate. The summary of the main themes is particularly helpful.
- There needs to be some discussion in this section of existence of opposing views to the ones presented. The wider context around the findings and any other research which may contradict these findings need to be explored, and linked back to the research gap that this study was filling.
- The limitations are clear, perhaps the strengths need to be better situated within the available literature and existing knowledge on this topic.
- Future directions for the research and practice are clear and meaningful.

References:

- Some inconsistencies for referencing (particularly of The Lancet - Lancet, T. as author of one editorial, please correct this in line with style guidelines). Please ensure consistency and revise these.

Recommendations:

- This article has an appealing focus on the articulation of dignity through Twitter during the start of the COVID-19 pandemic. It touches on key concepts around dignity and multiple disadvantage which were exacerbated during the pandemic. It would benefit from some restructuring (particularly in the results section, see comments) and a deeper critical engagement with concepts of dignity, gender, age, and the wider political and cross-national social contexts within which the texts studied were created. It would also benefit from a clearer explanation of the necessity of this study, and of the trends and oppositions in existing theoretical and empirical literature in this research area.  

Is the work clearly and accurately presented and does it cite the current literature?

No

If applicable, is the statistical analysis and its interpretation appropriate?

Not applicable

Are all the source data underlying the results available to ensure full reproducibility?

No

Is the study design appropriate and is the work technically sound?

Yes

Are the conclusions drawn adequately supported by the results?

Partly

Are sufficient details of methods and analysis provided to allow replication by others?

Yes

Reviewer Expertise:

Meiko Makita: social gerontology, feminist methodology, social media analysis, discourse analysis.                                                                                                                                               Hannah Loret: health, gender, qualitative research, interdisciplinary practice

We confirm that we have read this submission and believe that we have an appropriate level of expertise to confirm that it is of an acceptable scientific standard, however we have significant reservations, as outlined above.

## References

[1] : Hostility Toward Baby Boomers on TikTok. *Gerontologist* .2022;62(8) : 10.1093/geront/gnac020 1196-1206 10.1093/geront/gnac020 35106587 PMC9451021

[2] : The ‘Boomer remover’: Intergenerational discounting, the coronavirus and climate change. *The Sociological Review* .2022;70(1) : 10.1177/00380261211049023 74-91 10.1177/00380261211049023

[3] : Ethical approval: none sought. How discourse analysts report ethical issues around publicly available online data. *Research Ethics* .2021;17(3) : 10.1177/1747016120988767 275-297 10.1177/1747016120988767
